# Supplementary material for: α‐Synuclein strain homogeneity in multiple system atrophy subtypes
Source: Brain Pathol. 2026 Aug 14:e70134. Online ahead of print. doi: 10.1111/bpa.70134 (PMC13476587; doi:10.1111/bpa.70134)
Supplement: Supplementary file 1 [file BPA-9999-e70134-s001.pdf]

Supplemental Table S1. MSA patient sample details

Sample	Neuropathological diagnosis[1]	Clinical diagnosis	Age at death (yr)	Disease duration (yr)[2]	Sex	Postmortem interval (h)	Brain region(s)[3]	Used for inoculation studies?	Co-pathologies[4]
MSA-C_1	MSA-C	MSA	60	9	F	32	SN, TC, Cb	Yes	CVD
MSA-C_2	MSA-C	MSA-C	68	8	M	6	TC, Cb	Yes	
MSA-C_3	MSA-C	MSA	53	5	M	21	SN, TC, Cb	No	
MSA-C_4	MSA-C	MSA-C	69	7	F	84	SN, TC, Cb	No	CVD
MSA-C_5	MSA-C	MSA	68	6	M	12	SN, TC, Cb	No	ADNC (Braak stage II, Thal stage 3)
MSA-C_6	MSA-C	MSA	57	8	F	24	SN, TC, Cb	No	CVD
MSA-C_7	MSA-C	MSA	66	N/A	M	19	TC, Cb	No	LBD, ADNC (Braak stage I, Thal stage 3)
MSA-C_8	MSA-C	MSA	70	N/A	F	12	TC, Cb	No	Arteriolosclerosis
MSA-P_1	MSA-P	PD	76	7	M	24	SN, TC, Cb	Yes	CVD, ADNC (Braak stage V, CERAD B)
MSA-P_2	MSA-P	MSA	73	N/A	M	24	SN, TC, Cb	Yes	CVD, ADNC (Braak stage II, CERAD A, mild CAA)
MSA-P_3	MSA-P	MSA-P	62	13	M	36	SN, TC, Cb	No	CVD
MSA-P_4	MSA-P	PD	66	N/A	F	24	SN, TC, Cb	No	ADNC (Thal stage 4)
MSA-P_5	MSA-P	MSA-P	70	N/A	M	13	SN, TC, Cb	No	
MSA-P_6	MSA-P	MSA	63	4	F	20	SN, TC, Cb	No	ADNC (Braak stage II)
MSA-P_7	MSA-P	Progressive supranuclear palsy	75	3	F	12	TC, Cb	No	CVD

[1]MSA-C was diagnosed based on olivopontocerebellar atrophy whereas MSA-P was diagnosed based on striatonigral degeneration
[2]N/A: information not available
[3]SN: substantia nigra; TC: temporal cortex; Cb: cerebellum
[4]CVD: cerebrovascular disease; ADNC: Alzheimer's disease neuropathological changes; LBD, Lewy body disease; CAA: cerebral amyloid angiopathy

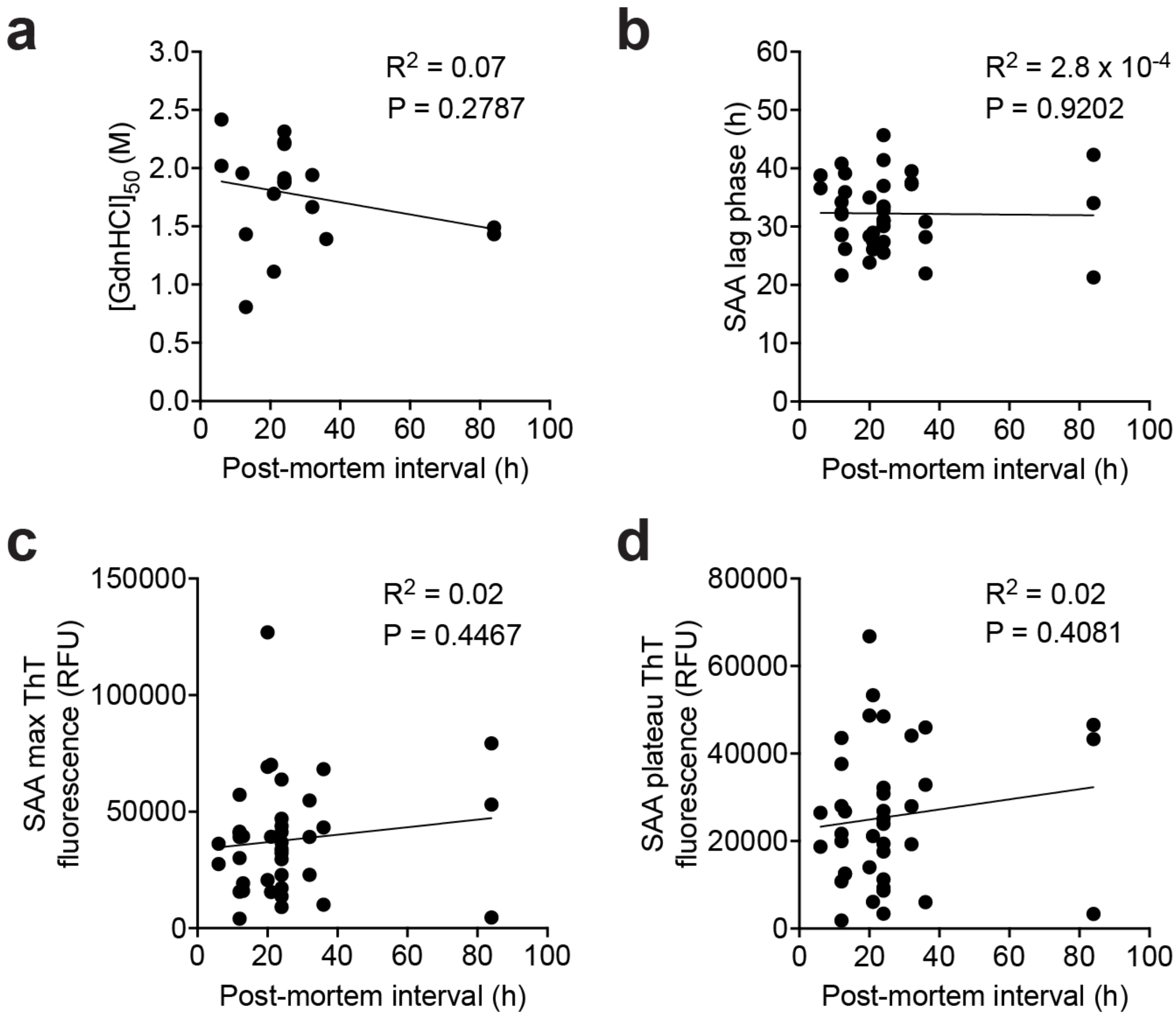

Supplemental Figure S1. The post-mortem interval has no effect on the conformational stability or the seed amplification assay attributes for α-syn aggregates in MSA brain homogenates. a) Correlation analysis between the post-mortem interval and the conformational stability ($[GdnHCl]_{50}$ values) of α-syn aggregates in MSA brain homogenates (n = 18). **b-d**) Correlation analysis between the post-mortem interval and the lag phase (b), maximum ThT fluorescence value (c), and plateau ThT fluorescence value (d) following SAA using MSA brain homogenates as the seed (n = 39). The R^2 and P values were calculated by performing Pearson correlation analysis.

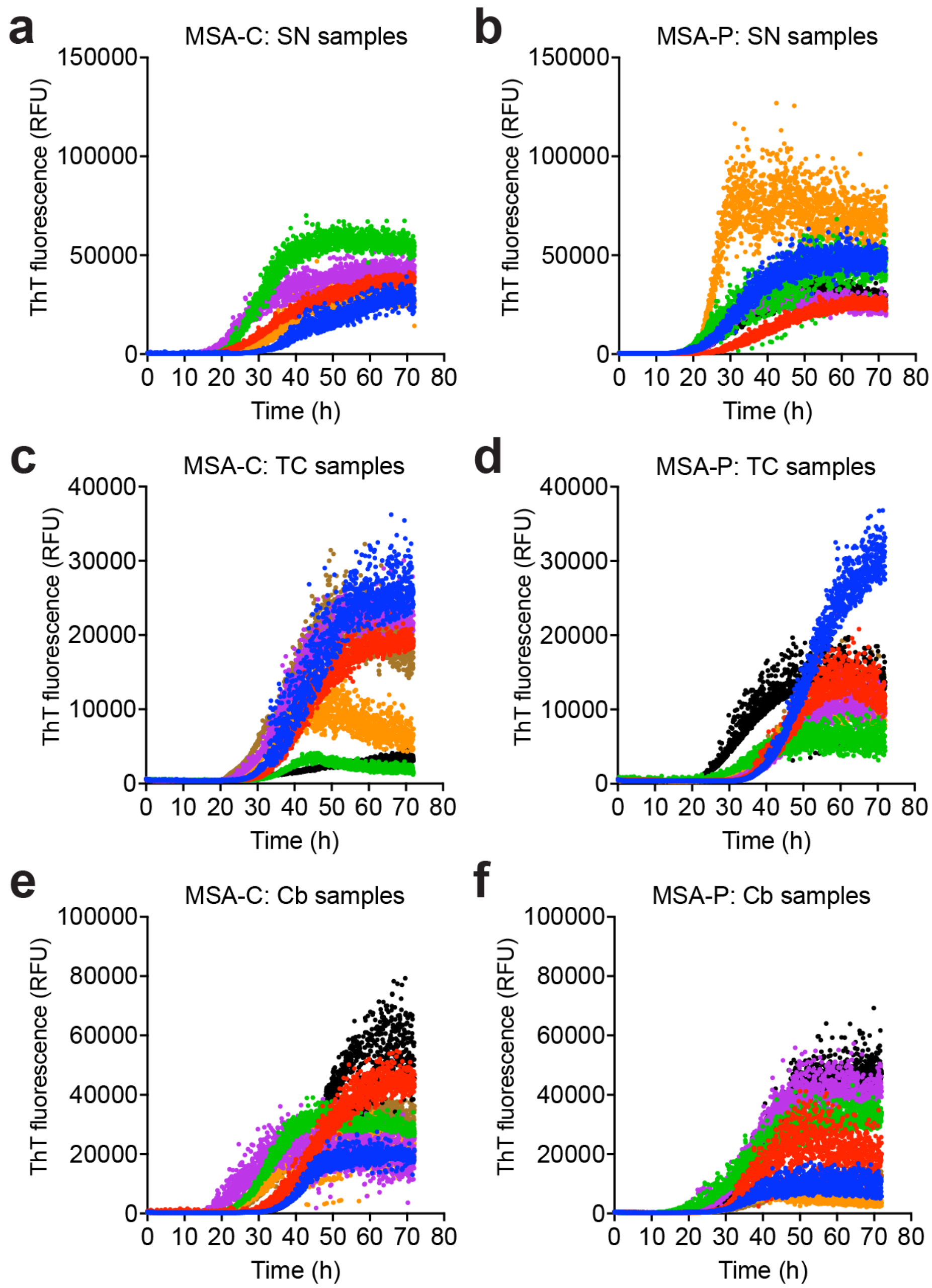

Supplemental Figure S2. α-Synuclein seed amplification curves for individual MSA-C and MSA-P patient samples. Individual ThT aggregation curves for SAAs using homogenates from the substantia nigra (a, b), temporal cortex (c, d), or cerebellum (e, f) of MSA-C (a, c, e) and MSA-P (b, d, f) patients. For SN samples, n = 5 (MSA-C) or n = 6 (MSA-P); for TC and Cb samples, n = 7 per MSA subtype. Each curve depicts the mean ThT fluorescence over time for 3-4 technical replicates per MSA sample.

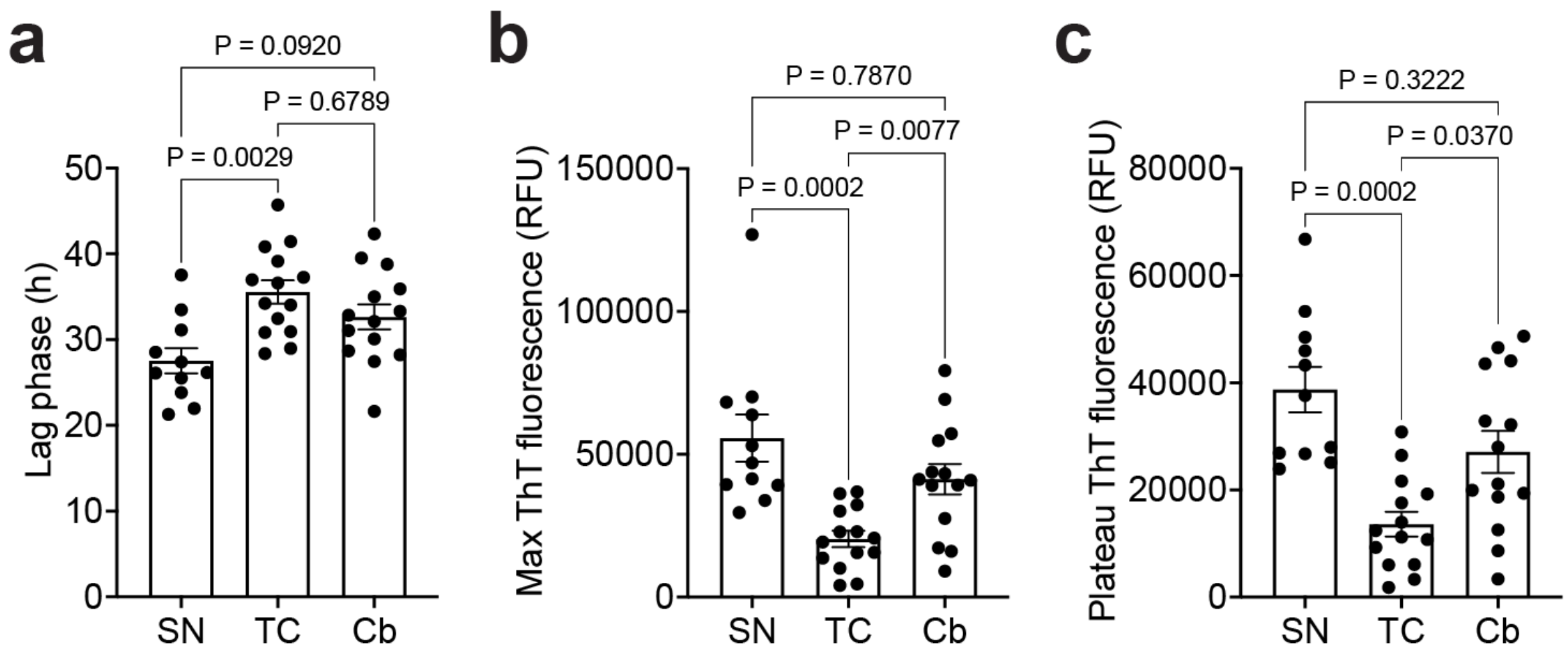

Supplemental Figure S3. Comparison of SAA attributes following amplification of α-syn aggregates from different MSA brain regions. Lag phase (a), maximum ThT fluorescence (b), and plateau ThT fluorescence (c) values following SAA using homogenates from either the substantia nigra (SN, n = 11), temporal cortex (TC, n = 14), or cerebellum (Cb, n = 14) of MSA patients. Graphs display mean ± s.e.m. Statistical significance was assessed using a Kruskal-Wallis test followed by Dunn's multiple comparisons test.

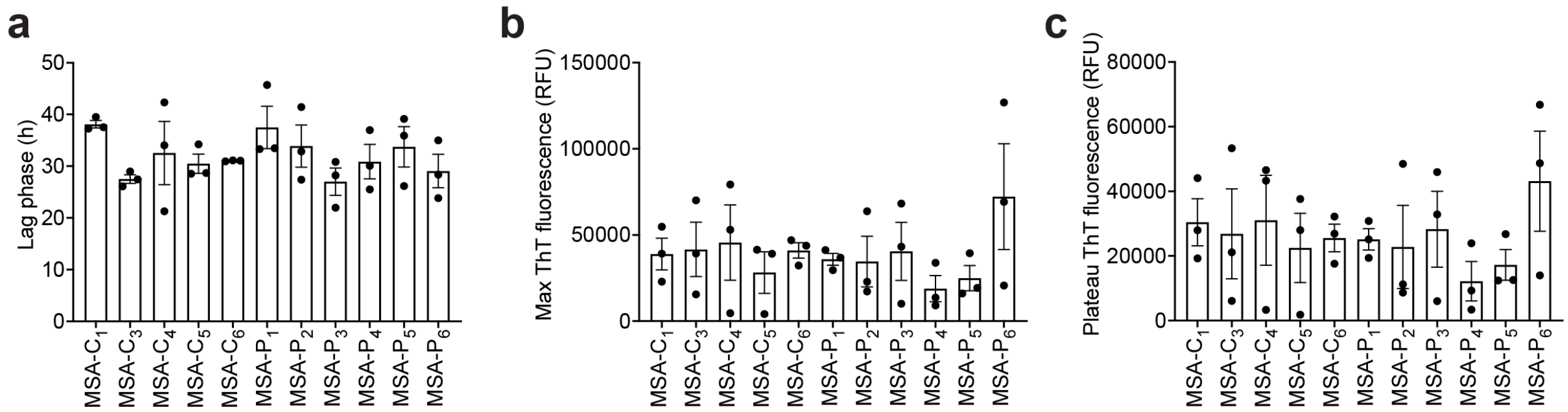

Supplemental Figure S4. Variation in SAA attributes within brain regions from individual MSA-C and MSA-P cases. Lag phase (a), maximum ThT fluorescence (b), and plateau ThT fluorescence (c) values following SAA using homogenates from individual MSA-C and MSA-P cases. For each case, graphs display mean ± s.e.m. for n = 3 brain regions (substantia nigra, temporal cortex, and cerebellum).

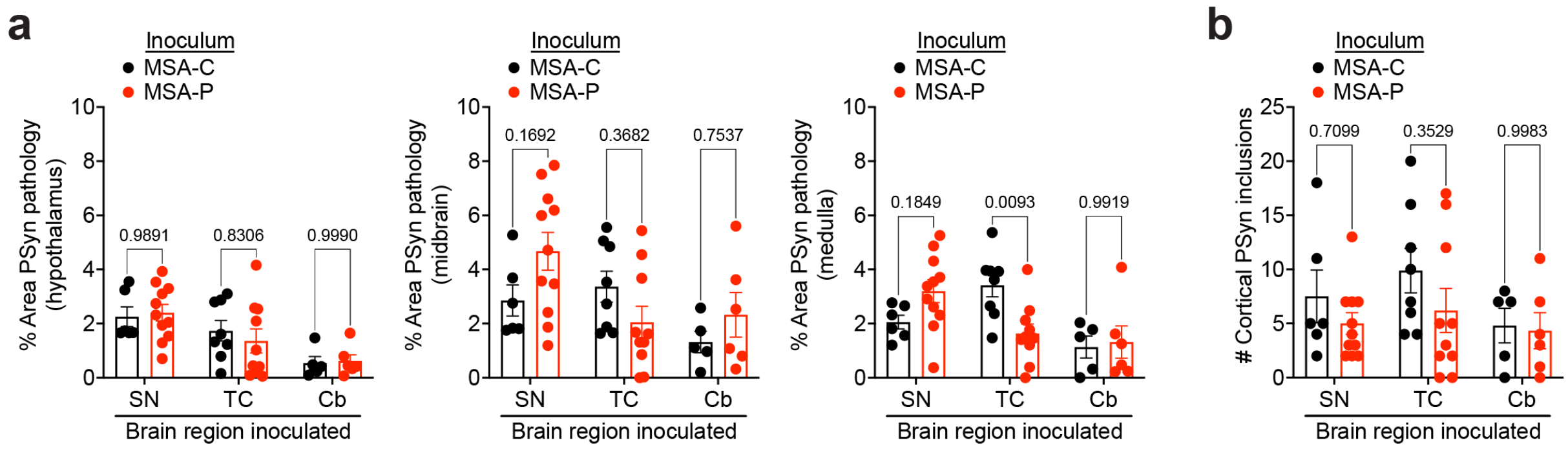

Supplemental Figure S5. Quantification of α-syn deposition in the brains of M83$^{+/-}$ mice inoculated with samples from different brain regions of MSA-C and MSA-P patients. a) Quantification of the percent area covered by PSyn staining in the hypothalamus (left graph), midbrain (middle graph), or medulla (right graph) from M83$^{+/-}$ mice inoculated with substantia nigra, temporal cortex, or cerebellar homogenate from MSA-C or MSA-P patients. **b**) Quantification of the number of cortical PSyn deposits in the brains of M83$^{+/-}$ mice inoculated with substantia nigra, temporal cortex, or cerebellar homogenate from MSA-C or MSA-P patients. For SN samples, n = 6 or 11 mice for MSA-C and MSA-P, respectively; for TC samples, n = 8 or 10 mice for MSA-C and MSA-P, respectively; for Cb samples, n = 5 or 6 mice for MSA-C and MSA-P, respectively. Graphs display mean ± s.e.m. Statistical significance was assessed by two-way ANOVA followed by Šidák's multiple comparisons test.

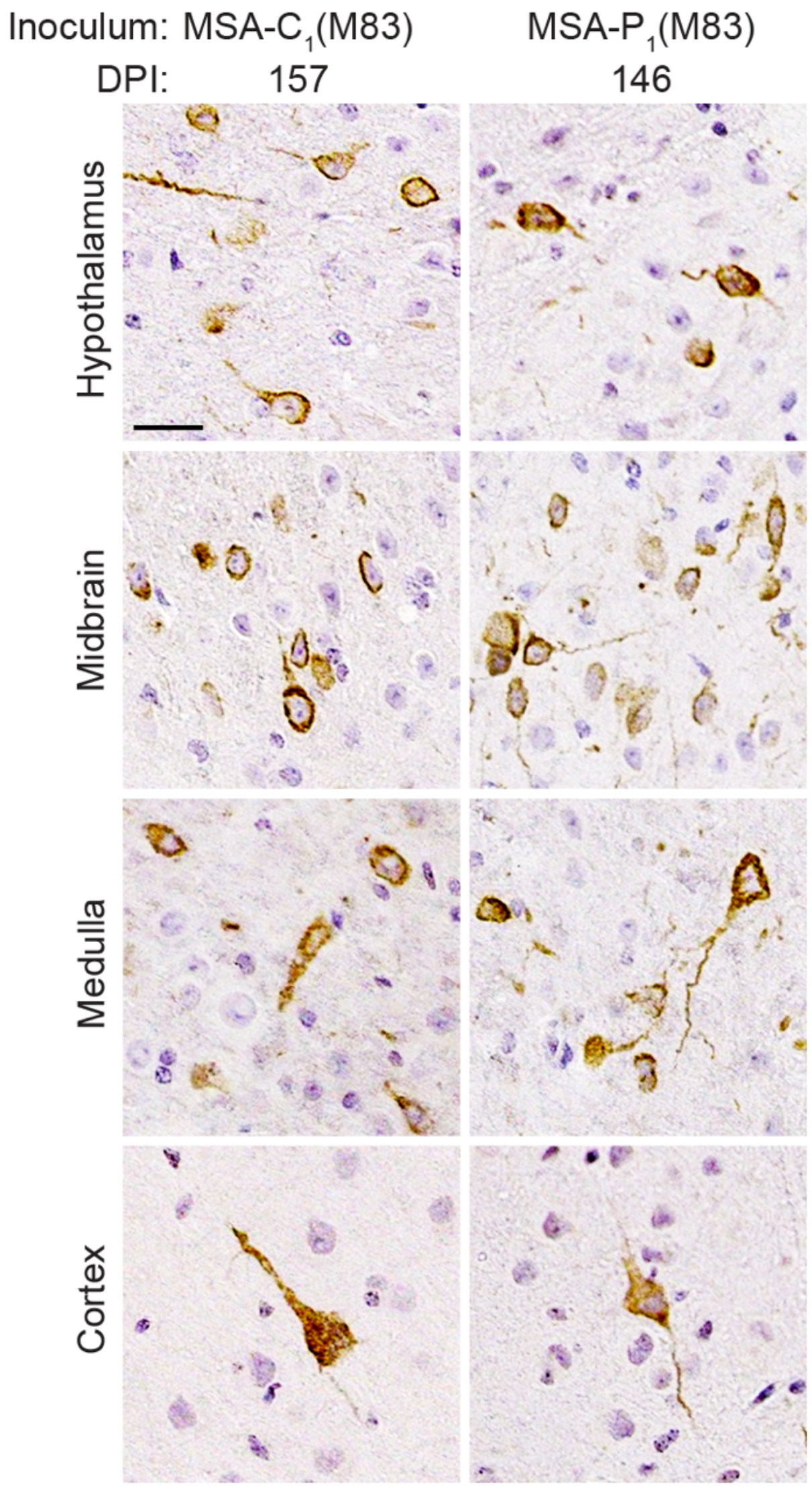

Supplemental Figure S6. Cerebral α-syn deposition in M83$^{+/-}$ mice inoculated with passaged MSA-C and MSA-P samples. Representative images of PSyn-stained sections from the hypothalamus, midbrain, medulla, and cortex of clinically ill M83$^{+/-}$ mice following inoculation with the indicated passaged MSA samples. PSyn was detected using the antibody EP1536Y. Scale bar = 20 μm (applies to all sections).
